# Annotations

**Published:** 1891-10-17

**Authors:** 


### Annotations.

**Mr. W. H. Smith.**

Mr. W. H. Smith, whose loss the Queen and the nation are now mourning, might have been the doctor of the House of Commons, and all its members his patients; so gentle was he with its refractory children, its impulsive and hysterical women, and its obstinate and self-willed men. Strange that among so many men of greater and more conspicuous gifts he should, by universal consent, have been considered so worthy of the place of honour. It is clear that even in the fast-moving England of to-day pushing adventurousness is not to have all its own way. Men prize still, and prize highly, plain honesty and staunch adherence to simple straightforwardness, a good-natured habit of taking hard blows without minding them very much, and a steady determination to keep the wheels of public business moving by alternate pushings behind, and promises of corn or carrots, rather than by a too demonstrative use of the whip. Mr. Smith was not a mere statesman; he was an example to all men. He showed how valuable a thing it is to do one's work well, and to take the reward which comes from good work well done, rather than to look upon the reward as the main result to be achieved, and to give the work a mere secondary place. Mr. Smith was especially an example to doctors. He did not rush his career, and most certainly they cannot rush theirs. If any man has to depend upon his work for his reward it is the doctor; and if any man has to wait with patience for results it is also the doctor. Still, as in the case of Mr. W. H. Smith, the reward comes in due time if the work be well done and the patience sufficiently enduring. It will surprise no one to learn that hospitals with other benevolent institutions shared liberally in Mr. Smith's great wealth. Among other charities to which he gave material support were the Brompton Consumption Hospital, the Royal Chest Hospital, City Road, the National Hospital for the Paralyzed, Hospital for Sick Children, the Orphan Working School, Commercial Travellers' Benevolent Institution, St. George's Hospital, King's College Hospital, City of London Truss Society, Newspaper Press Fund, the Mary Wardell Convalescent Home for Scarlet Fever, Female Mission to the Fallen, Home for Female Orphans, and the Metropolitan Drinking Fountain Association. A long list worthy of the man and of his sound and discriminating judgment.

**Golden Rules.**

As the principles of surgery become better understood its practice is better taught. The last thirty years have seen extraordinary progress in the comprehension of the physiological, pathological, and mechanical principles on which intelligent surgery is founded. The rule of thumb is absolutely and for ever out of date. Whilst experience is as valuable as it was a thousand years ago, experience alone, without an intelligent knowledge of principles, is like a man pursuing a path with which he may be quite familiar, but pursuing it always in a more or less dense fog. Thoroughly comprehended principles are the sunshine that clears the mist away and makes the whole path and the surrounding country plainly and distinctly visible. Whilst, however, we have constantly to insist upon principles, and indeed because we properly comprehend them, we know how to value clear and detailed rules. A little book has just been published by a hospital surgeon called "Golden Rules of Surgical Practice." It is intended for the use of dressers and junior house surgeons; but, if we mistake not, it will have a considerable sale among family practitioners as well. It contains but fifty-four small pages, and is one of those every-day reference books which should always be lying on the consulting room table. We have dipped into it more or less at random in several places, and it is a pleasure to record that at every place we found simple and intelligible rules which may properly be called "golden." For example, under the heading "Abscess," we find the three following, with many more of a like kind: "Never try fluctuation across a limb, but always along it"; "Do not forget that incisions in the neck and face should run parallel with the wrinkles and folds"; "Do not open an abscess anywhere near a large artery without first using a stethoscope, and then only by Hilton's method, that is, director and dressing forceps." Under the heading "Wounds," we read: "Never forget that the surgeon who neglects to suture a divided nerve or tendon commits the same mistake as he who neglects to reduce a fracture." And again, "Never forget that if an operation wound suppurates, the fault lies with the operator or his assistants." That is possibly a too comprehensive generalisation, though, of course, it is substantially true. Under the heading "General" it is thus laid down: "Remember three drugs are tolerated well in proportion to their need, viz., opium, mercury, and iodide of potassium." Also this, which is a golden rule that should be printed in letters a foot long, and hung up in a conspicuous place in every medical man's consulting room: "Do not form hasty opinions; and if you have formed a false opinion admit your error at once." The publishers of the excellent little book are Messrs. John Wright and Co., of Bristol.

**The Muzzle wanted again.**

Hydrophobia is beginning to present itself again in different parts of England. A month or two ago a patient died of it at Ealing, and the week before last a commercial traveller at St. Thomas's Hospital also succumbed to the disease. Whilst the muzzling order was in full operation cases of hydrophobia gradually and steadily diminished, until at last they ceased altogether. It may be taken as proved that the efficient muzzling of dogs infallibly results in the extirpation of hydrophobia. It cannot be considered that our legislators have behaved with any high degree of wisdom in the matter of unmuzzling. Is it permissible ever to use compulsion in the preservation of the public health? Apparently we in this country think it is; for we have to a large extent adopted the principle in order to secure the notification of infectious diseases; and in the matter of vaccination compulsion is absolute and universal. Now let us put these two things together, compulsory vaccination and the compulsory muzzling of dogs. So far as mere suffering is concerned—whether does the six-weeks-old vaccinated baby or the muzzled dog endure most? Granted that the prick of the vaccinating instrument produces little pain, it is not to be denied that the inflammation surrounding the developing and the fully matured pocks, and the elevation of temperature and general fever set up by and after the operation of vaccination, give rise to severe local pain and uneasiness, and to illness of a really distressing kind. All this we inflict on human babies, or if their parents object, we fine and imprison them without any hesitation in the interests of the public safety. But when it comes to the question of muzzling dogs we refuse to act at all until we are terrified into action by the universal spread of hydrophobia; and then when our muzzling order has proved its value by its complete success, we promptly set to work to undo all the good we had previously done. This may please certain people who have little mental capacity, and no capacity at all for considering the public safety. Such persons think much more of their own dog than of their neighbour's child. Well, but more intelligent and stronger-minded people should not yield to those who are merely sentimental and stupid. Every man of weight and judgment should put his foot down and insist that human beings shall be preferred to dogs. If the owners of the dogs shriek and behave like lunatics it may be well to consider the propriety of muzzling them too. So far as the dogs themselves are concerned, it is certain that in this country, where they are so numerous and of such various breeds, we do them a great service by muzzling them. When they are muzzled they cannot fight, and they cannot pick up all sorts of vile and poisonous garbage. The worst enemies our British dogs have are those foolish and inconsequent persons who object to the muzzle.

